# Complete sequence determination of a novel reptile iridovirus isolated from soft-shelled turtle and evolutionary analysis of *Iridoviridae*

**DOI:** 10.1186/1471-2164-10-224

**Published:** 2009-05-14

**Authors:** Youhua Huang, Xiaohong Huang, Hong Liu, Jie Gong, Zhengliang Ouyang, Huachun Cui, Jianhao Cao, Yingtao Zhao, Xiujie Wang, Yulin Jiang, Qiwei Qin

**Affiliations:** 1 State Key Laboratory of Biocontrol, School of Life Sciences, Sun Yat-sen University, 135 West Xingang Road, Guangzhou 510275, PR China; 2 Laboratory of Marine Biology, South China Sea Institute of Oceanology, Chinese Academy of Sciences, 164 West Xingang Road, Guangzhou 510301, PR China; 3 Shenzhen Exit & Entry Inspection and Quarantine Bureau, Shenzhen 518001, PR China; 4 State Key Laboratory of Plant Genomics, Institute of Genetics and Developmental Biology, Chinese Academy of Sciences, Beijing 100101, PR China

## Abstract

**Background:**

Soft-shelled turtle iridovirus (STIV) is the causative agent of severe systemic diseases in cultured soft-shelled turtles (*Trionyx sinensis*). To our knowledge, the only molecular information available on STIV mainly concerns the highly conserved STIV major capsid protein. The complete sequence of the STIV genome is not yet available. Therefore, determining the genome sequence of STIV and providing a detailed bioinformatic analysis of its genome content and evolution status will facilitate further understanding of the taxonomic elements of STIV and the molecular mechanisms of reptile iridovirus pathogenesis.

**Results:**

We determined the complete nucleotide sequence of the STIV genome using 454 Life Science sequencing technology. The STIV genome is 105 890 bp in length with a base composition of 55.1% G+C. Computer assisted analysis revealed that the STIV genome contains 105 potential open reading frames (ORFs), which encode polypeptides ranging from 40 to 1,294 amino acids and 20 microRNA candidates. Among the putative proteins, 20 share homology with the ancestral proteins of the nuclear and cytoplasmic large DNA viruses (NCLDVs). Comparative genomic analysis showed that STIV has the highest degree of sequence conservation and a colinear arrangement of genes with frog virus 3 (FV3), followed by Tiger frog virus (TFV), Ambystoma tigrinum virus (ATV), Singapore grouper iridovirus (SGIV), Grouper iridovirus (GIV) and other iridovirus isolates. Phylogenetic analysis based on conserved core genes and complete genome sequence of STIV with other virus genomes was performed. Moreover, analysis of the gene gain-and-loss events in the family *Iridoviridae *suggested that the genes encoded by iridoviruses have evolved for favoring adaptation to different natural host species.

**Conclusion:**

This study has provided the complete genome sequence of STIV. Phylogenetic analysis suggested that STIV and FV3 are strains of the same viral species belonging to the *Ranavirus *genus in the *Iridoviridae *family. Given virus-host co-evolution and the phylogenetic relationship among vertebrates from fish to reptiles, we propose that iridovirus might transmit between reptiles and amphibians and that STIV and FV3 are strains of the same viral species in the *Ranavirus *genus.

## Background

Iridoviruses are nuclear and cytoplasmic large DNA viruses (NCLDVs), which infect invertebrates and poikilothermic vertebrates, such as insects, fish, amphibians and reptiles, crustaceans and mollusks [1]. The serious systemic diseases caused by some members of the *Iridoviridae *family have made an important impact on modern aquaculture and wildlife conservation. The current members of the family *Iridoviridae *can be divided into five genera: *Ranavirus, Lymphocystivirus, Megalocytivirus, Iridovirus *and *Chloriridovirus *[2]. Typical characteristics of all iridoviruses include the icosahedral viral particles (~120 to 300 nm) present in the cytoplasm; also, the iridovirus genomes are circularly permuted and terminally redundant [3,4]. At present 13 iridovirus agents isolated from amphibians, fish and insects have been sequenced completely. These include Lymphocystis disease virus 1 (LCDV-1, genus *Lymphocystivirus*), Chilo iridescent virus (CIV, genus *Iridovirus*), Tiger frog virus (TFV, genus *Ranavirus*), infectious spleen and kidney necrosis virus (ISKNV, genus *Megalocytivirus*), Singapore grouper iridovirus (SGIV, genus *Ranavirus*), Frog virus 3 (FV3, genus *Ranavirus*), Lymphocystis disease virus China (LCDV-C, genus *Lymphocystivirus*), Grouper iridovirus (GIV, genus *Ranavirus*), Ambystoma tigrinum virus (ATV, genus *Ranavirus*), Rock bream iridovirus (RBIV, genus *Megalocytiviru*s), Red sea bream iridovirus (RSIV, genus *Megalocytiviru*s), Orange-spotted grouper iridovirus (OSGIV, genus *Megalocytivirus*) and Invertebrate iridescent virus 3 (IIV-3, *Chloriridovirus*) [5,6].

Soft-shelled turtle iridovirus (STIV), the causative agent of a novel viral disease called 'red neck disease' in the farmed soft-shelled turtle (*Trionyx sinensis*) in China was first reported in 1998 [7]. The virus could be propagated in several fish cell lines and caused an obvious cytopathogenic effect (CPE). To our knowledge, although several iridovirus-like agents from reptiles such as turtles have been isolated, no genomic information on a reptile iridovirus has been reported [8-11]. To facilitate understanding of the molecular mechanism of reptile iridovirus pathogenesis, we determined the complete genomic sequence of STIV and compared its genome structure with other sequenced iridoviruses to help determine its taxonomic position and evolutionary status.

## Results and discussion

### Features of the STIV genome

**Figure 1:**
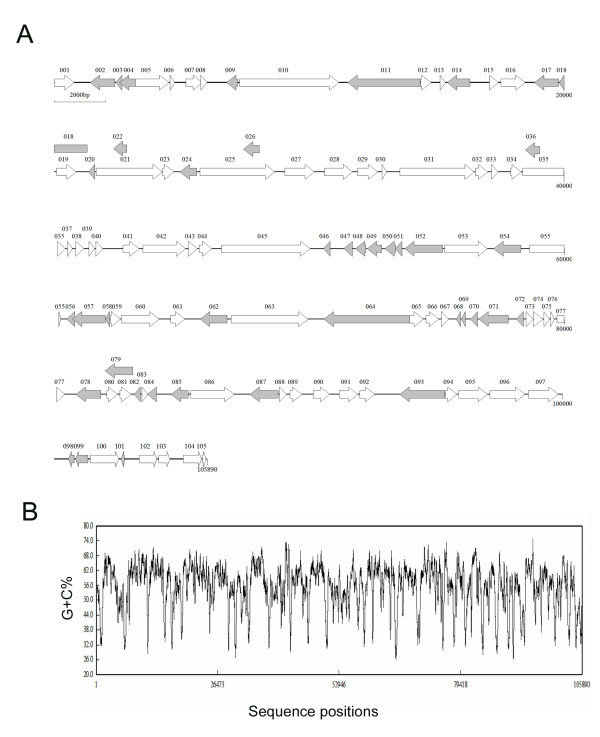
**Schematic organization of the STIV genome**. (A) Linear predicted open reading frame (ORF) map of the STIV genome. Predicted ORFs are represented by arrows indicating the approximate size and the direction of transcription based on the position of methionine initiation and termination codons. White arrows represent ORFs in the forward strand, whereas gray arrows identify those in the complement strand. (B) The G+C content of STIV genome. The graphic representation was calculated using the plot option in DNAMAN program and a window of 200 nucleotides. The kilobase scale is shown below the G+C plot.

The determination of the STIV complete genome sequence was carried out by 454 Life Sciences Technology as described [12]. About 2.1 million bp were sequenced, covering nearly 20-fold of the STIV genome sequence. The individual sequences were assembled into a continuous sequence using GS De Novo Assembler software (Roche). The results indicated that the complete STIV genome consists of 105 890 bp with 98.5% identity to the complete FV3 genome. The G+C content of STIV is 55.1% (Figure 1). Computer assisted analysis revealed 105 potential open reading frames (ORFs), which encode polypeptides ranging from 40 to 1,294 amino acids. The locations, orientations, sizes and BLASTP results for the putative ORFs are shown in Table 1. Forty-two individual putative gene products showed significant homology to functionally characterized proteins of other species. Forty-nine ORFs with unknown function have orthologs in other sequenced iridovirus genomes and 14 ORFs share no homology with other iridovirus genes. Seven ORFs (003L, 019R, 022L, 026L, 036L, 080R and 081R) that partially overlapped with others are not annotated as ORFs in the FV3 genome. The other seven ORFs (023R, 033R, 039R, 069L, 078R, 101L and 105R) have corresponding orthologs in the FV3 genome, but their annotations were missed in analysis [13]. The reconstructed common ancestor of the NCLDVs had at least 41 genes [14], whereas in the STIV genome only 20 putative protein products shared homology with the ancestral proteins of NCLDVs, including proteins involved in viral DNA replication, transcription, virion packaging and morphogenesis (see Additional File 1). In addition, a few noncoding regions were identified in the STIV genome and this feature is similar to FV3. In these regions, 20 microRNAs were predicted and are described in detail below.

**Table 1:** Properties of ORFs within the STIV genome

					Best Match homolog				
						
ORF	Nucleotide position	No. of amino acids	Molecular mass (kDa)	Conserved domain or signature (CD/Prosite accession no.)	E-value	Identity (%)	Accession no.	Species	Predicted structure or function
001R	16–786	256	29.67	Poxvirus Late Transcription Factor (pfam04947)	1e-148	99	YP_031579	FV3	putative replicating factor
002L	2385-1414	323	35.01	DUF230, Poxvirus proteins of unknown function (pfam03003)	2e-157	97	YP_031580|	FV3	Virion-associated membrane protein
003L^a^	2668-2423	81	8.82						
004L	3261-2563	232	25.74		1e-90	99	YP_003773	ATV	
005R	3191–4507	438	48.29		0.0	98	YP_031581	FV3	
006R	4547–4729	60	6.51		1e-18	100	YP_031582	FV3	
007R	5162–5818	218	24.82	US22, herpes virus early nuclear protein (pfam02393)	3e-100	92	YP_031583	FV3	orf250-like protein
008R	6008-5757	83	9.69		2e-37	100	YP_031584	FV3	
009L	7177-6769	142	15.18		8e-55	91	ABB92275	TFV	
010R	7277–11161	1294	140.99	DNA-directed RNA polymerase subunit alpha (PRK08566,)	0.0	99	YP_031586	FV3	DNA-dependent RNA polymerase II large subunit
011L	14356-11510	948	106.45	Helicase conserved C-terminal domain (pfam00271)	0.0	99	YP_031586	FV3	D6/D11 like helicase
012R	14372–14785	137	14.88		2e-70	100	YP_031588	FV3	
013R	15135–15347	70	7.88		5e-24	98	YP_031589	FV3	
014L	16306-15413	297	32.66		7e-146	99	YP_031590	FV3	
015R	17072–17428	118	13.31		6e-44	99	YP_031592	FV3	
016R	17524–18471	315	35.37	ABC_ATPase, Poxvirus A32 protein (pfam04665, cd00267)	0.0	98	AAL77796	TFV	A32 virion packaging ATPase
017L	19770-18835	311	34.00		5e-157	95	YP_003857	ATV	
018L	21315-19807	502	53.47		0.0	99	YP_031595	FV3	
019R^a^	20090–20869	259	28.11						
020L	21591-21352	79	8.35		3e-28	98	YP_031596	FV3	
021R	21643–24240	865	94.42	2-cysteine adaptor domain(pfam08793)	0.0	92	ABB92284	TFV	
022L^a^	22859-22326	177	18.78						
023R	24251–24697	148	16.03		5e-60	92	ABB92285	TFV	
024L	25593-24934	219	25.37		8e-112	98	YP_031599	FV3	
025R	25723-28650	975	108.95	D5 N terminal like (pfam08706)	0.0	99	YP_031600	FV3	putative D5 family NTPase/ATPase
026L^a^	28049-27423	208	21.58		9e-40	75	YP_164148	SGIV	
027R	29058–30206	382	42.61		0.0	97	YP_031601	FV3	
028R	30604–31701	365	41.04		0.0	98	YP_031602	FV3	
029R	31895–32680	261	39.50		2e-128	100	YP_031603	FV3	P31k protein
030R	32858–33034	58	6.07		2e-23	90	AAD38359	FV3	truncated putative eIF-2alpha-like protein
031R	33565–36477	970	107.18	Putative lipopolysaccharide modifying enzyme (smart00672)	0.0	98	YP_031605	FV3	tyrosine kinase
032R	36526–37014	162	18.21		2e-89	99	YP_031606	FV3	
033R	37151–37411	86	9.44						
034R	37905-38324	139	15.15		2e-72	98	ABB92294	TFV	
035R	38374–40308	644	71.50	Rho termination factor (pfam07498)	0.0	86	ABB92295	TFV	neurofilament triplet H1 like protein
036L^a^	39047-38472	191	20.01						
037R	40391–40582	63	6.64		1e-14	98	YP_031611	FV3	
038R	40726–41046	106	11.39		6e-50	99	YP_031612	FV3	L-protein-like protein
039R	41140–41460	106	10.20						
040R	41515–41790	91	9.14						
041R	42588–43229	213	23.62	catalytic domain of ctd-like phosphatases (smart00577)	3e-109	99	YP_031615	FV3	putative NIF/NLI interacting factor
042R	43370–45067	565	62.28	ribonucleotide-diphosphate reductase subunit alpha (PRK09102)	0.0	99	YP_031616	FV3	ribonucleoside diphosphate reductase alpha subunit
043R	45173–45523	116	12.72		3e-41	98	YP_031617	FV3	putative hydrolase
044R	45592–46095	167	18.05		4e-64	88	YP_031618	FV3	
045R	46477–49974	1165	129.13		0.0	99	YP_031619	FV3	orf2-like protein
046L	50766-50509	85	9.36		3e-30	100	YP_031620	FV3	
047L	51634-51308	108	12.02		1e-44	96	YP_003844	ATV	
048L	52171-51761	136	15.55		2e-72	100	YP_031623	FV3	
049L	52803-52225	192	21.62	translation initiation factor IF-2 (PRK05306)	2e-35	77	YP_003846	ATV	neurofilament triplet H1 protein
050L	53344-52928	138	15.54		3e-66	100	YP_031625	FV3	
051L	53598-53347	83	9.56		8e-35	100	YP_031626	FV3	
052L	55205-53706	499	55.48	SAP domain (pfam02037)	2e-149	78	YP_003850	ATV	
053R	55285–56970	561	61.62		0.0	99	YP_031629	FV3	
054L	58294-57227	355	39.35	3-beta hydroxysteroid dehydrogenase(pfam01073)	0.0	100	ABI36881	RGV	3beta-hydroxysteroid dehydrogenase
055R	58633–60201	522	54.73	L1R_F9L, Lipid membrane protein of large eukaryotic DNA viruses (pfam02442)	0.0	100	YP_031631	FV3	myristylated membrane protein
056L	60755-60435	106	11.50						
057L	61963-60668	431	47.29	DEXH-box helicases (cd00269)	0.0	99	YP_031633	FV3	A18 like helicase
058L	62120-61971	49	5.19		2e-09	93	YP_003822	ATV	
059R	62157–62561	134	15.23		6e-66	97	ABB92316	TFV	
060R	62602–64098	498	53.60		0.0	98	YP_031636	FV3	putative phosphotransferase
061R	64549–65103	184	20.49		3e-92	97	ABB92319	TFV	
062L	66787-65729	352	40.04		0.0	98	YP_031638	FV3	
063R	66947–69988	1013	114.52	DNA polymerase family B (pfam00136)	0.0	99	YP_031639	FV3	DNA polymerase
064L	74278-70613	1221	133.21	RNA polymerase beta subunit (cd00653)	0.0	99	YP_031641	FV3	DNA-dependent RNA polymerase II, subunit II
065R	74004–74540	178	19.64		2e-19	38	YP_164170	SGIV	
066R	74657–75151	164	17.38	dUTPase (COG0756)	2e-84	100	AAZ22692	RGV	dUTPase
067R	75261–75548	95	10.38	CARD, Caspase recruitment domain (pfam00619)	3e-35	94	YP_031643	FV3	CARD-like protein
068L	76015-75851	54	4.95		1e-08	98	YP_031644	FV3	
069L	76210-76061	49	5.12						
070L	76693-76400	97	10.81		5e-23	96	YP_031645	FV3	
071L	77911-76748	387	43.88	Ribonucleotide Reductase beta subunit (cd01049)	0.0	99	YP_031646	FV3	ribonucleotide reductase small subunit
072L	78502-78236	88	9.28		8e-28	79	YP_003808	ATV	
073R	78619–78894	91	9.72		6e-31	100	YP_031648	FV3	
074R	78903–79277	124	13.37		2e-44	100	YP_031649	FV3	
075R	79316–79549	77	8.34		1e-27	98	YP_031650	FV3	
076R	79593–79727	44	4.89						
077R	79834–80316	160	17.24		2e-78	95	YP_003804	ATV	
078L	81742-80768	324	36.13	Zinc finger C2H2 type domain signature (PS00028)	2e-178	98	YP_031652	FV3	ring finger protein
079L	83005-81917	362	38.14		5e-167	100	YP_031653	FV3	
080R^a^	81987–82415	142	14.81						
081R^a^	82500–82943	147	15.30						
082L	83316-83062	84	9.26	Possible membrane associated motif in LPS-induced tumor necrosis factor (smart00714)	7e-30	98	YP_031654	FV3	LPS-induced tumor necrosis factor alpha
083R	83379–83600	73	7.99		2e-31	95	YP_031655	FV3	
084L	83944-83597	115	12.84		6e-56	100	YP_031656	FV3	VLTF2-like late transcription factor
085L	85203-84529	224	25.60		5e-119	93	ABB92336	TFV	
086R	85303–87021	572	63.50		0.0	98	YP_031658	FV3	putative ATPase dependent protease
087L	88760-87645	371	40.36	Ribonuclease III C terminal domain (cd00593)	0.0	100	YP_031659	FV3	ribonuclease III
088R	88816–89094	92	10.51	C2C2 Zinc finger (smart00440)	1e-41	98	YP_031660	FV3	transcription elongation factor IIS
089R	89223–89696	157	17.65		5e-87	98	YP_031661	FV3	immediate early protein ICP-18
090R	90146–90790	214	24.73	Site-specific DNA methylase (COG0270)	2e-122	100	YP_031662	FV3	cytosine DNA methyltransferase
091R	91177–91914	245	26.05		5e-133	99	YP_031663	FV3	proliferating cell nuclear antigen
092R	91989–92576	195	22.12	Deoxyribonucleoside kinase (cd01673)	1e-106	99	YP_031664	FV3	thymidine kinase
093L	95345-93564	593	64.26		0.0	97	ABB92341	TFV	
094R	95378–95830	150	16.53	Erv1/Alr family (pfam04777)	4e-83	99	YP_031667	FV3	thiol oxidoreductase
095R	95899–97044	381	43.28		3e-140	92	YP_031668	FV3	
096R	97137–98528	463	49.92	Iridovirus major capsid protein (pfam04451)	0.0	99	YP_031669	FV3	major capsid protein
097R	98652–99839	395	45.57	T4 RNA ligase (pfam09511)	0.0	98	YP_031670	FV3	immediate early protein ICP-46
098L	100767-100552	71	7.63		3e-12	98	YP_031671	FV3	
099L	101295-100828	155	17.85		2e-79	100	YP_031673	FV3	
100R	101389–102480	363	40.63	Xeroderma pigmentosum G N- and I-regions (cd00128)	0.0	99	YP_031674	FV3	FLAP endonuclease
101L	102699-102571	42	4.31						
102R	103281–103952	223	24.28		7e-123	98	YP_031675	FV3	
103R	104035–104448	137	15.29		5e-73	99	YP_031676	FV3	Bcl-2-like protein
104R	104973–105677	234	26.9	herpes virus US 22 like protein (pfam02393)	4e-47	51	YP_031583	FV3	
105R	105716–105856	46	5.73						

Note. FV3, Frog virus 3; TFV, Tiger frog virus; ATV, Ambystoma tigrinum virus; SGIV, Singapore grouper iridovirus; RGV, Rana grylio virus.

^a^putative overlapped ORFs in STIV genome.

### Repetitive sequences

Repetitive sequences are not only found in eukaryotic genomes [15], but have also been identified in large DNA viruses, where they are involved in genome replication and gene transcription [16,17]. Similar to other iridoviruses, the STIV genome contains 21 repeat sequences (Table 2). Interestingly, a 34 tandem repeated CA dinucleotide called microsatellite or simple sequence repeat (SSR) was closely associated with a predicted gene encoding for a ring finger protein (ORF078L) in the STIV genome. Such a repeat sequence has only been reported in the FV3 genome, but not in other sequenced iridoviruses or mammalian large DNA viruses. These SSRs could serve to modify viral genes involved in gene regulation, transcription and protein function and modification in their function mainly depends on the number of repeats [18]. The biological functions of the repeat sequences and the CA dinucleotide microsatellite in STIV remain to be characterized.

**Table 2:** Sets of repeat sequences in STIV genome

Location	size (bp)	Copy number	Matches (%)	G+C content (%)
800 – 845	15	3.1	96	30
1004 – 1190	64	2.9	97	32
1499 – 1578	9	8.9	95	63
11243 – 11282	14	2.9	100	22
22236 – 22276	15	2.7	100	50
28871 – 28952	30	2.7	100	26
38641 – 39108	222	2.1	100	50
39783 – 39831	21	2.3	100	66
41855 – 41886	16	2.0	100	43
43198 – 43222	6	4.2	100	68
45776 – 45812	18	2.1	100	55
49952 – 50070	39	3.1	100	36
52268 – 52597	18	18.3	100	55
54757 – 54890	39	3.4	100	56
60328 – 60387	16	3.8	97	21
65561 – 65592	15	2.1	100	33
80609 – 80676	2	34.0	100	50
93066 – 93178	30	3.8	98	51
95254 – 95296	18	2.4	100	78
96781 – 96922	21	6.8	100	65
99849 – 99882	11	3.1	100	44

### DNA replication and repair

STIV encodes a protein (ORF063R) similar to family B DNA polymerases, which contains a nucleotide-polymerizing domain fused to an N-terminal exonuclease domain. In eukaryotes and prokaryotes, DNA polymerase is an essential replication enzyme and is able to proofread misincorporated nucleotides as well as replicate DNA [19]. Besides these functions, the poxvirus DNA polymerases could also play critical roles in catalyzing concatemer formation and promoting virus recombination [20,21]. Some viruses, such as baculoviruses and poxviruses, not only exploit the host cell proliferating cell nuclear antigen (PCNA) proteins to contribute to viral DNA replication [22], but also encode *PCNA*-like genes by themselves [23,24]. A homolog of PCNA was identified in STIV. STIV also encodes a homologue of the poxvirus D5 family proteins (ORF025R) that contains a unique D5N domain and belongs to the helicase superfamily III within the AAA+ ATPase class [25]. The highly conserved D5 protein is required for the viral DNA replication or lagging-strand synthesis [26].

Other putative proteins encoded by STIV with known or presumed functions in viral DNA replication, recombination and repair included thymidine kinase (ORF092R), virion packaging ATPase (ORF016R), helicase (ORF057L) and tyrosine kinase (ORF031R) as well as FLAP endonuclease (ORF100R) with a conserved nuclease domain (N- and I- regions). The FLAP endonuclease homologs are not only present in STIV and other iridoviruses, but also in the poxvirus, ascovirus and mimivirus [14]. Interestingly, FLAP endonuclease homologs have been identified in herpesviruses and shown to destabilize preexisting host mRNAs in infected cells [27]. Thus, the protein product of ORF100R might function in STIV virogenesis.

### Proteins involved in transcription

The gene products involved in transcription include two DNA-dependent RNA polymerase subunits (DdRP, ORF010R and ORF064L), transcription factor-like proteins (ORF001L), transcription elongation factor S-II/TFIIS (ORF088R) and a putative NIF/NLI interacting factor containing a CTD phosphatase domain (ORF041R). The DNA-dependent RNA polymerases (DdRPs) are multifunctional enzymes and exist ubiquitously in prokaryotes, eukaryotes and cytoplasmic DNA viruses [28,29]. The putative protein encoded by ORF088R contains a C2C2 zinc finger domain and is homologous to the TFIIS, which is ubiquitous in many organisms and plays an important role in transcript elongation [30,31]. Virally encoded TFIIS regulate the elongation potential of the viral RNA polymerase during vaccinia virus infection [32].

### Nucleotide metabolism

Four proteins involved in nucleotide metabolism were predicted in the STIV genome, including the large and small subunits of the ribonucleotide reductase (RNR, ORF042R and ORF071L respectively), deoxyuridine triphosphate nucleotidohydrolase (dUTPase, ORF066R) and RNase III (ORF087L). Viral RNR is either required for virus growth or is involved in anti-apoptosis functions during viral pathogenesis [33,34]. A putative dUTPase homolog encoded by ORF066R contains five conserved motifs and a conserved Tyr residue as the substrate binding site. dUTPase is an essential enzyme and plays multiple cellular roles [35]. In cells infected with Epstein-Barr virus, virally encoded dUTPase homologs function as highly specific enzymes for efficient replication, or serve to upregulate several proinflammatory cytokines [36,37].

STIV ORF087L also contains a well-conserved RNase III catalytic domain that is required for the cleavage of double stranded (ds)RNA templates [38]. Nearly all STIV encoded nucleotide metabolism enzymes have orthologs in other large DNA viruses. This is consistent with the view that the frequent acquisition of nucleotide metabolism enzymes during DNA virus evolution appears to reflect specific adaptations of viruses for the different types of cells in which they propagate [22].

### Structural proteins

Despite the emerging information about iridovirus genomes, there has been little focus on the roles of structural proteins in viral pathogenesis. Three putative structural proteins were examined in the STIV genome. ORF096R encodes a major capsid protein 463 amino acids long that shares 99% identity to FV3. Similar to the MCP gene, the two other genes, ORF002L and ORF055R, are also highly conserved in all sequenced iridovirus genomes [5]. ORF002L encodes a putative membrane protein with a poxvirus conserved region and a C-terminal transmembrane domain. In addition, ORF055R is a myristylated membrane protein homolog with two adjacent transmembrane domains and a conserved sequence M-G-X-X-X-(S/T/A) for N-terminal glycine myristylation. The myristylated membrane protein encoded by vaccinia virus plays a role in virus assembly [39]. The roles of the two putative membrane proteins of STIV during viral infection need to be evaluated.

### Virus-host interactions

In addition to the essential genes required for virus replication, STIV also contains several putative genes involved in host-virus interactions, especially in immune evasion. STIV ORF054R shares 40% identity with the vaccinia virus 3-beta-hydroxysteroid oxidoreductase-like protein (3-β-HSD), which has been suggested to contribute to virulence by suppressing inflammatory responses [40]. In addition, three proteins that might be involved in apoptotic signaling have also been identified: ORF067R encodes a protein containing caspase recruitment domain (CARD) and ORF082L encodes a protein sharing sequence homology with the lipopolysaccharide induced tumor necrosis factor-alpha (LITAF) of viruses and eukaryotes [41,42]. There is also a Bcl-2-like protein (ORF103R) containing BH1, BH2 domains and a typical 'NWGR' signature motif. Bcl-2 homologs are also found in herpesviruses, poxvirus, African swine fever virus (ASFV) and adenoviruses [43]. Considering that several iridovirus agents can induce apoptosis during infection, and that virally induced apoptosis aids the progression of replication and dissemination [44,45], these apoptosis-regulating genes might manipulate the balance of life and death in STIV infected cells. In addition, the virally encoded eIF-2α decoy could inhibit eIF-2α phosphorylation and block interferon action during virus infections. Interestingly, STIV ORF030R also displays a truncated eIF-2α-like protein as well as FV3 ORF026R, which is different from the complete eIF-2α homologs conserved among eukaryotes and other viruses, suggesting that STIV and FV3 are likely isolates of the same viral species.

### Noncoding RNAs

MicroRNAs (miRNAs) are key regulators of gene expression in higher eukaryotes. Recently, miRNAs have been identified from viruses with double-stranded DNA genomes. The computational method has been applied successfully to predict miRNAs encoded by herpes simplex virus 1 and human cytomegalovirus [46,47]. We applied the same algorithm to the STIV genome and searched for 21-nucleotide (nt) sequences with hairpin-structured precursors. Twelve precursor sequences encoding 20 miRNA candidates were identified in the STIV genome (Table 3). MicroRNAs of mammalian viruses play important roles during infection, such as repressing host immune responses and apoptosis, and regulating gene expression [48,49]. Whether the potential miRNAs are functional in STIV needs further investigation.

**Table 3:** Sequences of predicted STIV pre-miRNAs and miRNAs and their genomic locations

Precursor no.	Predicted pre-miRNA sequence, 5' to 3' (mature miRNA sequence highlightened in italic)	STIV sequence coordinates
1	GGUGUAACAUC***UCAAGAUACGAUGGAUCUAUG***AGAGAGACUAAAAAUGUGGACAACCUUUCAGACUAUUAUCUUGAGAGAAUAUAUCUU	14907–14995
2	AAAAGUUUCCGAGAUGGUAAAGACUCUGAGAUAAUAUCGAGAGAAUAAAGACUC***UUUCAGAGAUAAUAUCUUACG***AUGUUGUACCACCUCAUU	18571–18663
3	AACAACG***UCUUGAGAUACUAUUAUCUUA***AGAUACUAUUAUC***UUAAGAUACUAUUAUCUUAAG***AUACUUUC	60321–60390
4	ACAAACUGGUGA***UAUAUUCUUUCAGAAGAUAUU***CUCUGGGAGAG***UAUCUUUCAGAAGAUUAUAUC***UCAGAAAGUUUUAGG	65173–65252
5	GUCUAGAAAUAUUAUUGAGGGUAUCUUACAAUAUUAGUAAAGAUAUCUUCUGAAAGAGUAUCU***UACUAUAGUAGUACAGUAUCU***UACAAUAGAGAGAUCU	87293–87392
6	UUAGGUCUGGAUAUUA***UCUCUGAAAGAACGUCUUAGG***AUAAAAUC***UUAGGAUAUUCUUUCAGAAGA***UUUCUAGGAUAAGA	42283–42362
7	UGACGGAGGGUUGUUCCACUCCACGGGGGCUUUGGGACACUCUACCUGAACCC***UGGGUGGAGACCACUCUUUGU***A	195–121
8	CGGUGCGA***UCGGUGUACACACAAGUGAUG***GACACACCACACAGG***UCCAGCACGUGUGUACACCAG***AGGUAAUUUUCUUAA	4966-4887
9	UAGAGAUGGUA***AUAUCUUAAGAUAAUAGUAUC***GAGAUGGUAAUAUCUUAAGAUAAUAGUA***UCGAGAUGGUAAUAUCUUAAG***AUAUUUAGU	28954-28865
10	GCGAGAUACUU***UGUGAGAGAUAUCUUACGAUA***GUAAUAGUCUUGCGAGAGAAUAUCUUCUGAAAGAGAUUAUAUCUGAAAGAGAU***UACGUCUUAAGAUAUCUUACA***CAUCACUCUUGUCCU	84184-834064
11	GGUUUCGGCGGCAAUAAGGCGAG***UCUCAACAUUAAACCCCAUAC***AAAGUCUACGGUCUC***UGUAUGAGGAAUGUUGGGAC***ACUUGCGCUUGUAACAACGCUUGCAGUCU	100334-100217
12	GGGACCCUUUAAAUCAGAAAGGAUAACACCAG***UGUAAACAUAAGUCAUAUGCC***UGUGUUGGUUCUCACAGG***UGUGUUACUUAUGUUUACACU***GGUCUUAGCCUUGCUGGA	104919-104810

### Global pairwise alignment and core gene order comparisons

**Figure 2:**
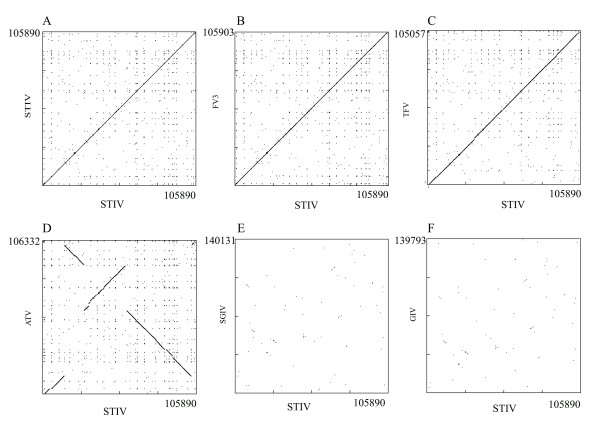
**DNA dot plot analysis of the STIV genome (horizontal axis) with itself and other members belonging to the ranaviruses (axis)**. The vertical axes represent the genomes of (A) STIV, (B) FV3, (C) TFV, (D) ATV, (E) SGIV and (F) GIV. The complete genomic sequences were aligned using the DNAMAN program and both strands of DNA were aligned for the dot matrix plot. Solid lines show the high level of sequence similarity.

DNA dot matrix (Pustell DNA matrix) analyses of STIV complete genomic sequence with the FV3, TFV, ATV, SGIV, GIV (Figure 2), LCDV-1, LCDV-C, ISKNV and CIV genome sequences (results not shown) revealed that STIV has a high degree of sequence conservation and colinearity with FV3. A slight break was also present when STIV is compared to TFV. Interestingly, STIV have little colinearity with the fish iridoviruses, SGIV and GIV, the second group of genus *Ranavirus*.

**Figure 3:**
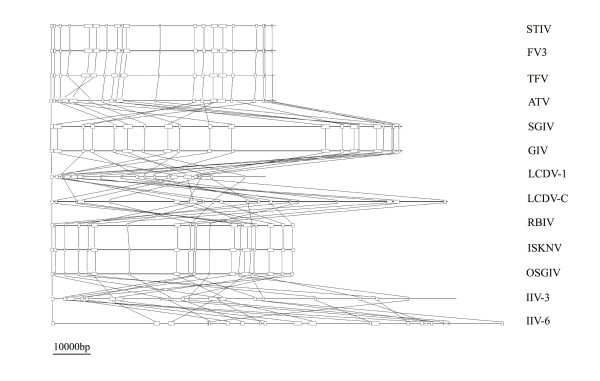
**The genomic arrangement of 20 conserved genes in the family *Iridoviridae***. Genes are indicated by black outline boxes. The MCP genes were designated as the starting point for all iridovirus genomes and genome names are listed on the right. Horizontal distances are shown proportional to base pair distances and the vertical lines indicate the conserved genes in different iridovirus isolates. The following are the conserved genes according to their order in the STIV genome: major capsid protein (096R); immediate-early protein ICP-46 (097R); FLAP endonuclease (100R); putative replicating factor (001R); DNA-dependent RNA polymerase II largest subunit (010R); D6/D11-like helicase (011L); A32 virion packaging ATPase (016R); unknown protein (021R); unknown (024L); D5 family NTPase (025R); NIF/NLI interacting factor (041R); myristylated membrane protein (055R); phosphotransferase (060R); DNA polymerase (063R); DNA-dependent RNA polymerase subunit II (064L); ribonucleotide reductase small subunit (071R); Ribonuclease III (087L); proliferating cell nuclear antigen, PCNA (091R); thymidine kinase (092R) and thiol oxidoreductase (094R).

We also examined the arrangement of 20 conserved genes, including the major capsid protein and other proteins involved in genome replication, transcription and modification. Given that the origin of virus genome replication is unclear, the MCP gene was chosen as the starting point for all iridovirus genomes. As shown in Figure 3, STIV has a gene order in common with FV3 and TFV, but shows obvious differences from ATV, SGIV and GIV. In addition, the orders of these genes are significantly discriminative among different genera. The presence of inversion in ATV and different gene arrangements are consistent with the high recombination frequency in iridoviruses.

### Phylogenetic analysis

**Figure 4:**
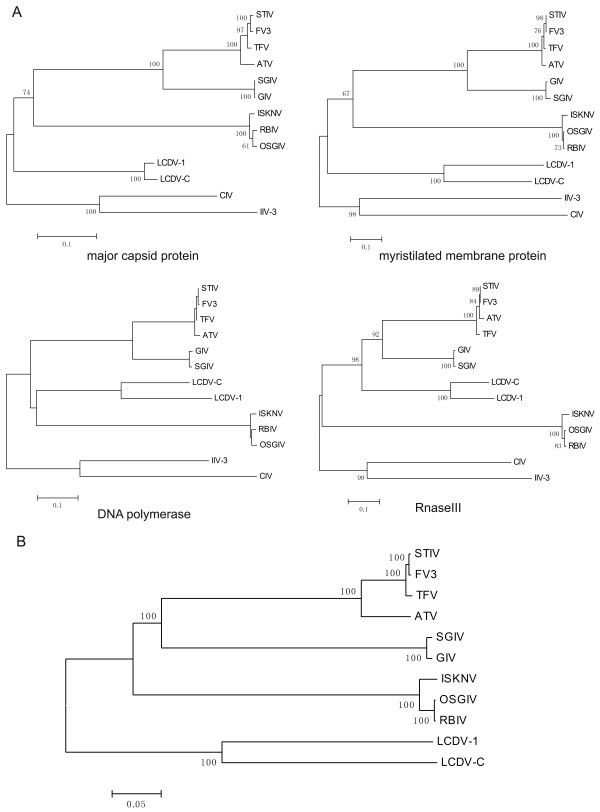
**Phylogenetic analysis of STIV with other iridovirus isolates based on four conserved core genes and the complete genome sequence**. (A) Complete amino acid sequences of major capsid protein (ORF096R), myristilated membrane protein (ORF055R), DNA polymerase (ORF063R) and ribonuclease III (ORF087L) of STIV, FV3, TFV, ATV, SGIV, GIV, LCDV-C, LCDV-1, CIV, IIV-3, ISKNV, RBIV and OSGIV were aligned using Clustal-X and parsimony bootstrap trees generated using PHYLIP. Numbers above branches indicate bootstrap support values based on 100 replicates. (B) Unrooted phylogenetic tree of vertebrate iridoviruses based on the complete genomic sequences. Alignments were made using the MAFFT 6 program and a dendrogram was constructed using the MEGA4 program.

To test the phylogenetic relationship of STIV with other members of iridoviruses, the full-length protein sequences encoded by four conserved core genes, including the major capsid protein (MCP), a myristilated membrane protein, ribonuclease III and DNA polymerase (DNA pol) were used for phylogenetic analysis. The alignments were performed using ClustalX and the unweighted parsimony bootstrap consensus tree was obtained by heuristic search with 100 bootstrap replicates. As shown in Figure 4A, the results from four phylogenetic trees provided consistent evidence that STIV is most closely related to FV3, the typical species of the genus *Ranavirus*, followed by TFV, ATV, SGIV and GIV.

Furthermore, given the significant difference in the genome length between vertebrate and invertebrate iridoviruses, a phylogenetic analysis based on the complete genomes of 11 sequenced vertebrate iridovirus isolates was performed. The results further suggested that STIV is most closely related to FV3 (Figure 4B). Given the nature of virus-host coevolution and the phylogenetic relationships among vertebrates from fish to reptiles, we propose that the iridovirus might transmit between reptiles and amphibians, and that STIV and FV3 are strains of the same viral species belonging to the *Ranavirus *genus of family *Iridoviridae*. Interclass infections of iridovirus have been observed by *in vivo *and *in vitro *studies on sympatric species of fish and amphibians that can be infected by the same virus [50]. Whether the STIV infects frogs and FV3 infects turtles are questions that need to be evaluated.

### Gene gain and loss in the Iridoviridae family

**Figure 5:**
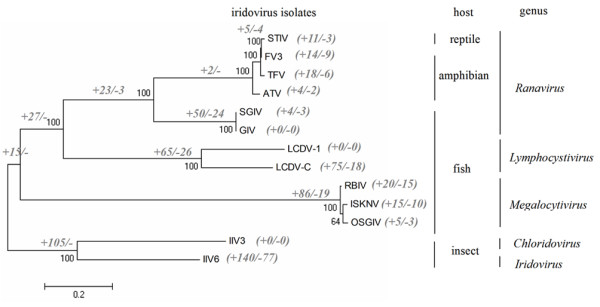
**Phylogeny of the iridovirus based on concatenated protein sequences and the gene gain and loss events**. The host of the virus and the virus classification are indicated on the right. Bootstrap values are shown in black next to the nodes and the numbers of gene gain (+) and loss (--) events along each branch are indicated in grey.

During virus-host coevolution, gene gain and loss are likely to have host-specific effects. The acquired genes could contribute to the evasion of host defenses, while the lost genes may coincide with either the loss of an antigenic signal to the host cell immune system or the gain of virulence [51,52]. To better understand the evolution of gene content in the *Iridoviridae *family, we analyzed the gene gain and loss events among the 13 sequenced iridovirus agents. According to our strict homology definition, only 11 clusters of orthologous groups (COGs) contained a homolog from all the iridovirus isolates. Several previously defined conserved core genes were excluded, including the putative replication factor and proliferating cell nuclear antigen (PCNA)-like proteins. These genes shared additional homology characteristics such as a predicted conserved domain, but showed poor alignment scores. We generated a phylogenetic tree based on these 11 concatenated proteins showing the number of genes gained and lost at each branch. As shown in Figure 5, although our mapping of gene gain and loss assumes that gene loss could occur throughout the tree, reptile ranavirus and amphibian ranavirus (+2/-) have less gene gain-and-loss events than fish ranavirus (+50/-24), fish lymphocystivirus (+65/-26), fish megalocytivirus (+86/-19) and insect iridovirus (+105/-). The variance among ranaviruses supported the point that SGIV and GIV were classified into the second *Ranavirus *group. Moreover, both STIV and FV3 gained five and lost four genes compared with TFV during evolution, again suggesting that STIV shares the highest identity with FV3. In addition, a number of COGs were only present within a specific genus. Tumor necrosis factor receptor (TNFR) homologs or TNFR-associated proteins were gained in fish iridovirus and lost in amphibian and reptile iridoviruses, while DNA topoisomerase II, NAD-dependent DNA ligase, SF1 helicase, inhibitor of apoptosis protein (IAP) and baculovirus repeated open reading frame (BRO protein) were lost in vertebrate iridoviruses. These genes might have contributed greatly in favoring adaptation to different natural host species during iridovirus-host co-evolution.

## Conclusion

In summary, the present study provided the complete genome sequence of turtle iridovirus. The phylogenetic tree and dot plot analyses suggested that STIV, a novel reptile iridovirus isolate, and FV3 are strains of the same virus species belonging to the genus *Ranavirus *in the family *Iridoviridae*. The genome data will not only contribute to better understanding the reptile iridovirus pathogenesis, but also shed light on the evolution of the different iridovirus isolates.

## Methods

### Virus propagation and genome DNA preparation

The virus strain used for genome sequencing was STIV (strain 9701) isolated from diseased red-neck turtle (Trionyx sinensis) in China [7]. Fathead minnow (FHM) cells were cultured in Minimum Essential Medium (MEM, Gibco/Invitrogen) containing 10% fetal bovine serum (FBS, Gibco). When STIV-infected FHM cells exhibited 80% CPE, cells were collected and frozen at -20°C. The frozen cells were thawed, and cell debris was removed by centrifugation at 4 000 × g for 30 min at 4°C and the supernatant containing STIV was ultracentrifuged in a Beckman (rotor type, SW41) at 28 000 rpm (~130 000 × g) for 1 h at 4°C. The pellet was resuspended in 1 ml of PBS and further centrifuged using discontinuous sucrose gradient (20, 30, 40, 50 and 60%) centrifugation at 28 000 rpm (~130 000 × g) for 1 h. The virus particle band was collected and used to prepare the STIV genomic DNA using phenol-chloroform extraction as described [53].

### DNA sequencing

Sequencing of STIV genome was carried out using a pyrosequencing platform, the Genome Sequencer 20 (GS20) System (454 Life Science Corporation, Roche). Briefly, after the quality of STIV genome DNA had been assessed by agarose gel electrophoresis and analysed by Agilent bioanalyzer (Agilent Technologies, Santa Clara, CA, USA), ~10 μg samples were sheared by nebulization into 300–500 bp fragments. The whole genomic library was amplified using GS20 emPCR kits and sequenced with the 454 Life Science GS 20 instrument according to the manufacturer's recommendations. The GS De Novo Assembler software generates a consensus sequence of the whole DNA sample by assembly of *de novo *shotgun sequencing reads into contigs and subsequent ordering of these contigs into scaffolds. The average reading frame length was about 100 bp with 20-fold coverage of the whole genome. To fill the gaps, 16 oligonucleotide primers were used to amplify by polymerase chain reaction (PCR) directly from the genome DNA and the corresponding PCR products were sequenced using an automated ABI 3730 apparatus (Applied Biosystems, Shanghai, China).

### Genome structure prediction

Nucleotide and amino acid sequences were analyzed using the DNASTAR software package (Lasergene, Madison, WI, USA). The genomic organization was drawn using the DNAMAN program. Nucleotide sequence and protein database searches were performed using the BLAST programs at the NCBI website . The whole genome sequence was also submitted to  (Softberry Inc., Mount Kisco, NY, USA) for identification of all putative ORFs. For more refined analyses, conserved motifs and domains and putative functions of deduced STIV proteins composed of 40 or more amino acids with homologies to other proteins in sequence databases were identified using several online programs as follows: for conserved motifs and domains,  and  were used; for transmembrane domain predictions,  was used. DNA repetitive sequences were detected computationally using REPuter and a tandem repeats finder [54]. The STIV microRNA prediction was carried out as described [47].

### Iridovirus phylogeny

To analyze the evolutionary position of STIV in the family *Iridoviridae*, four conserved iridovirus genes, which are also present in other large DNA viruses, were evaluated using the PHYLIP program based on the amino acid alignment. Multiple alignments of proteins and nucleotide sequences were generated using the MAFFT 6 and ClustalX programs [55,56]. In addition, a phylogenetic tree was constructed using MEGA version 4 with complete genomic sequences corresponding to the available sequencing data of iridoviruses.

### Gene gain and loss events in the Iridoviridae family

All the putative iridovirus genes were obtained from NCBI databases and the all-against-all BLASTP similarity search was performed. The different iridovirus genes were regarded as COGs based on protein sequence similarity. The homologs were determined if one hit the other in the BLASTP search with an e-value ≤ 10^-5 ^and the maximal produced alignments covered at least 60% of the longer protein, while the homologous proteins from multiple copies of a gene in one genome were counted only once. Eleven sets of COGs were aligned independently using the ClustalX alignment program, then the alignments were concatenated into a single alignment and a neighbor-joining (NJ) tree was constructed using MEGA version 4. Gene gain and loss events were processed with PAML software package and assigned to branches in the phylogenetic tree [57].

### Nucleotide sequence accession number

The complete STIV genome sequence has been deposited in GenBank under accession No. EU627010. The nucleotide sequences of other iridoviruses can be found in GenBank and the accession numbers were listed as follows: FV3, AY548484; TFV, AF389451; ATV, AY150217; GIV, AY666015; SGIV, AY521625; LCDV-1, L63545; LCDV-C, AY380826; ISKNV, AF371960; RBIV, AY532606; OSGIV, AY894343; IIV-6, AF303741 and IIV-3, DQ643392.

## Authors' contributions

YHH and XHH purified the STIV virus, prepared the viral genome DNA, performed the bioinformatics analysis and drafted the manuscript. JG, ZLOY, HCC, JHC, YTZ and XJW participated in primer design, PCR amplification and bioinformatics analysis. HL, YLJ and QQW contributed to the experimental design and manuscript editing.

## Supplementary Material

Additional file 1 **Ancestor proteins of large DNA viruses that present or absent in STIV genome**. In the STIV genome, only twenty putative protein products shared homology with the ancestral proteins of NCLDVs, including proteins involved in viral DNA replication, transcription, virion packaging and morphogenesis. Click here for file
