# Supplementary material for: Complete sequence determination of a novel reptile iridovirus isolated from soft-shelled turtle and evolutionary analysis of Iridoviridae
Source: BMC Genomics. 2009 May 14;10:224. doi: 10.1186/1471-2164-10-224 (PMC2689277; doi:10.1186/1471-2164-10-224)
Supplement: Additional file 1 — Ancestor proteins of large DNA viruses that present or absent in STIV genome. In the STIV genome, only twenty putative protein products shared homology with the ancestral proteins of NCLDVs, including proteins involved in viral DNA replication, transcription, virion packaging and morphogenesis. [file 1471-2164-10-224-S1.doc]

Additional file 1.

Ancestor proteins of large DNA viruses that present or absent in STIV genome

| Ancestral genes of NCLDV that present in STIV | Ancestral genes of NCLDV that absent in STIV |
| --- | --- |
| D5 like ATPase (ORF025R) | RNA polymerase, Subunit 5 |
| Fmaily B DNA polymerase (ORF063R) | RNA polymerase, Subunit 10 |
| VLTF2 Transcription factor (ORF084L) | Topoisomerase II |
| A32 virion packaging ATPase (ORF016R) | Thymidylate kinase |
| Transcription elongation factor IIS (ORF088R) | Thymidylate synthase |
| RNA polymerase, largest subunit (ORF010R) | AEP-type Primase |
| RNA polymerase, Subunit 2 (ORF064L) | A7L like transcription factor |
| Thymidine kinase (ORF092R) | Bro-N domain protein |
| Thiol oxidoreductase (ORF094R) | KilA-N domain protein |
| Proliferating cell nuclear antigen (ORF091R) | Capping enzyme |
| Ribonucleotide reductase, small subunit (ORF071L) | E1L like polyA polymerase |
| Ribonucleotide reductase, large Subunit (ORF042R) | E10R like thiol reductase |
| dUTPase (ORF066R) | SW12/SNF2 ATPase |
| Major capsid protein (ORF096R) | H1L like phosphatase |
| Myristoylated virion protein (ORF055R) | SMT4 like thiol peptidase |
| Virion-associated membrane proteins (ORF002L) | Ankyrin repeats |
| Hydrolase (ORF043R) | ATP dependent DNA ligase |
| D6/D11 like helicase (ORF011L) | RuvC like holloday junction resolvase |
| A18 like helicase (ORF057L) | F10 like protein kinase |
| FLAP endonuclease (ORF100R) | I8 like helicase |
|  | A2L like transcription factor |
